# SARS-CoV-2 infection after COVID-19 vaccinations among vaccinated individuals, prevention rate of COVID-19 vaccination: A systematic review and meta-analysis

**DOI:** 10.1016/j.heliyon.2024.e30609

**Published:** 2024-05-01

**Authors:** Dagne Deresa Dinagde, Bekam Dibaba Degefa, Gemeda Wakgari Kitil, Gizu Tola Feyisa, Shambel Negese Marami

**Affiliations:** Departments of Midwifery, College of Health Sciences, Mattu University, Mettu, Ethiopia

**Keywords:** SARS, COVID reinfection, Meta-analysis, COVID-19 vaccination, Post COVID-19

## Abstract

**Background:**

The global concern regarding protection against the COVID-19 variants through pre-existing antibodies from vaccination or previous infection is evident. Reports from around the world indicate that a considerable number of healthcare professionals/individuals experience re-infection despite being vaccinated. Moreover, several studies have highlighted cases of symptomatic SARS-CoV-2 re-infection, specifically among individuals who have been vaccinated. Understanding the factors that contribute to these re-infections is crucial for implementing effective public health measures and enhancing vaccination strategies.

**Method:**

A comprehensive search was conducted between January 1, 2021, and February 14, 2024, using various reputable sources such as PubMed, Google scholar, Medline, EMBASE, CINAHL, and others. The search aimed to retrieve relevant research on topics related to “world nations” and phrases like “COVID-19 vaccination breakthrough infection,” “SARS re-infection after COVID-19 vaccination,” “COVID-19 vaccine complication,” “post COVID-19 vaccination symptoms,” and specific nation names. The data obtained from the databases underwent extraction and quality assessment using the Newcastle-Ottawa Scale (NOS) and the Preferred Reporting Items for Systematic Review and Meta-Analyses. Data analysis was performed using STATA^17^MP software, and measures such as the I^2^ test statistic and Egger's test were used to assess heterogeneity and publication bias. The findings were presented using forest plots, displaying the odds ratio (OR) and 95 % confidence interval (CI).

**Result:**

This review and meta-analysis comprised a total of 15 articles, or a total sample size of 342,598. The pooled prevalence of SARS-CoV-2 after vaccination of COVID-19 was 9 % (95CI 7%–11 %) of population globally. This implied that reduced the overall attack rate of COVID-19 by 91 % after vaccination. The highest pooled estimated of SARS-CoV-2 infection after COVID -19 Vaccinations was seen among developing nations, 20 % (95 % CI: 5%–36 %).The pooled odds ratio showed that a significant association was found between SARS-CoV-2 infection after COVID-19 vaccination and older age (OR = 2.04; 95%CI: 1.10–2.98) and comorbidity (OR = 3.25; 95%CI: 1.04–5.47).

**Conclusion:**

It is important for policymakers to prioritize continuous monitoring and surveillance of SARS-CoV-2 infection rates among vaccinated individuals globally, as there is a significant estimate of the combined prevalence of post-COVID-19 vaccine SARS-CoV-2 infections.

## Highlights

• SARS-CoV-2 infection after COVID -19 Vaccinations was prevalent among developing nations.
• The introduction of vaccination reduced the overall attack rate of COVID-19 by 91 %.
• Individuals aged fifty or older were more prone to SARS CoV-2 infection.
• Policymakers should prioritize on surveillance of SARS-CoV-2 infection rates.

## 1 Introduction

Despite being vaccinated against COVID-19, there have been instances where individuals have been re-infected with the SARS-CoV-2 virus, which is a cause for concern. This phenomenon of SARS re-infection among vaccinated individuals has been observed in certain cases, highlighting the need to understand the underlying factors and implications [1,2]. It is essential to comprehend the factors that contribute to these re-infections to uphold effective public health measures and improve vaccination strategies. Investigating the prevalence, characteristics, and potential consequences of SARS re-infection can offer valuable knowledge for policymakers and healthcare professionals to mitigate risks and enhance protection among vaccinated populations [3].

Based on the analysis of the complete genetic sequence of SARS-CoV-2, including early human cases and samples from various regions, it has been determined that the virus originated from bat populations in an ecological setting. The available evidence strongly indicates that SARS-CoV-2 has a natural animal origin and is not a product of laboratory manipulation or construction. Numerous researchers have examined the genomic characteristics of SARS-CoV-2 and have found no supportive evidence to suggest that it is a laboratory-engineered virus [4]. As of December 2023, the global COVID-19 pandemic had resulted in approximately 701,748,397 confirmed cases and caused the loss of 6,968,845 lives worldwide. In East Africa, a total of 1,734,021 cases and 30,162 deaths were documented during the same period. Among the East African countries, Ethiopia reported the highest number of cases, with 501,117 infections, while Eritrea had the lowest number of cases, with 10,189 infections, by the end of December 2023 [5]. The pandemic has caused a startling loss of life on a global scale and poses an unparalleled threat to food systems, public health, and the workplace [6]. The global pandemic has had a profound impact on both the economy and society, resulting in significant disruptions. A substantial number of individuals, potentially reaching tens of millions, face the imminent risk of extreme poverty. Furthermore, the current estimate of approximately 690 million malnourished individuals may increase by up to 132 million by the end of the year [7].

Although COVID-19 had put negative impact in all world population, due to advanced technology and science, vaccine development had seen only in developed nations in order to stop the pandemic's spread [8,9]. Approximately 4.96 billion individuals worldwide have received at least one dose of the COVID-19 vaccine, equating to a rate of 63.69 per 100 people. Furthermore, 56.16 per 100 people have been fully vaccinated with the Eastern Mediterranean region had the highest proportion of individuals who received at least one dose (84.18 per 100 people) [10]. Nevertheless, the protective efficacy of vaccines diminishes over time and the levels of antibodies decline significantly within a few months following vaccination. Numerous instances of post-vaccination infections have been documented, indicating breakthrough cases. Through viral gene sequencing, it has been observed that some of these individuals were infected with distinct strains of the virus [11]. The incidence of SARS infection following COVID-19 vaccination was ranged from 0.05 % in California, united states [12] to 17.9 % in Saudi Arabia [13].

Conducting a thorough analysis of the occurrence of SARS-CoV-2 infections after COVID-19 vaccination and identifying the factors that contribute to re-infections among vaccinated individuals on a global scale would greatly assist researchers, policymakers, and health authorities in developing effective interventions and monitoring strategies for vaccinated individuals. However, comprehensive reviews focusing on COVID-19 vaccination-related SARS-CoV-2 infections and their predictors are currently lacking. This review aims to consolidate available evidence regarding the prevalence of SARS-CoV-2 infections following COVID-19 vaccination and the factors that influence such infections among vaccinated individuals in a global context.

### 1.1 Specific objectives

❖ What is the level of post COVID-19 vaccination SARS-CoV-2 infections among vaccinated clients?
❖ What are the predictors of post COVID-19 vaccination SARS-CoV-2 infections among vaccinated?

## 2 Methods and materials

### 2.1 Reporting

The review's findings were reported in accordance with the Preferred Reporting Items for Systematic Review and Meta-Analysis (PRISMA) guideline [14]. However, it is important to note that the review was not registered on PROSPERO, a prospective registry for systematic reviews and meta-analyses, as mentioned in the “limitations of the study” section.

### 2.2 Search strategies

A comprehensive search of multiple databases, namely PubMed, Embase, Science Direct, Cochrane Library, African Journals Online, Google Scholar, and Web of Science, was conducted to ensure a thorough exploration of relevant literature. The search strategy involved using specific terms such as “COVID-19 vaccination breakthrough infection” or “SARS re-infection after COVID19 vaccination”, or “COVID-19 vaccine complication”, “post COVID-19 vaccination symptoms” and “name of nation” and “name of nations” “factors”, “post COVID-19 reinfection [Mesh Terms]” and “breakthrough” and “name of nation”. The reference lists of every study that was included were examined in order to find other studies that were missed. A search of the literature was done between January 1 and February 14, 2024.

## 3 PECO

The review employed the Population, Exposure, Comparison, and Outcomes (PECO) framework to guide the analysis. The PECO statement for this review included the following elements: Population (all individuals who received COVID-19 vaccination in any countries); Exposure (factors influencing post COVID-19 vaccination SARS symptoms; Comparison (control groups outlined in individual studies; and Outcome: occurrence of post COVID-19 vaccination SARS infection) (Yes/No).

### 3.1 Eligibility criteria

The inclusion criteria for this systematic review and meta-analysis involved using both the CoCoPop (condition, context, and population) and PECO (population, exposure of interest, and outcome or response) approaches. Studies that did not align (qualitative) with these approaches were deemed irrelevant and excluded. The evaluation encompassed various study designs, including longitudinal, cohort and cross-sectional studies that reported on SARS-CoV-2 after of COVID-19 vaccination in the general population. The review included studies published in English up until January 12, 2024. All articles underwent quality evaluation using the Newcastle-Ottawa Scale (NOS) critical appraisal instrument and were deemed of sufficient quality. Excluded from the study were reviews, commentary and studies lacking transformable raw data.

### 3.2 Data extraction

A piloted Microsoft Excel spreadsheet was utilized for data extraction, with four authors (DD, SHN, GW, and BD) independently extracting data from the included studies. The spreadsheet included information such as the author's name, publication year, study year, study location, sample size, and the proportion or magnitude of post COVID-19 vaccination SARS-CoV-2 infections. In cases where there were discrepancies among the reviewers, these were resolved through discussion and a reassessment of each study. Articles that provided incomplete abstracts or texts, or reported outcomes outside the predetermined parameters of interest, were excluded. Once agreement was reached, the data extraction process was concluded.

### 3.3 Data quality assessment

The Newcastle-Ottawa Scale (NOS) quality assessment tool was used to assess the quality of included studies based on the three components [15]. The assessment instrument consisted of three components to evaluate the methodological rigor, comparability, and results/statistical analysis of each primary study. The first component used a scale of 1–5 to assess the methodological rigor of each study. The second component, graded with two stars, focused on concerns related to comparability among the studies. The last component, scored with three stars, evaluated the results and statistical analysis of each original study. The Newcastle-Ottawa Scale (NOS) encompassed three categorical factors, with a maximum score of 10. Each study was assigned a quality rating based on the scoring criteria: a score of seven or higher was considered “good” quality, a score of five to six was deemed “fair” quality, and a score of four or lower was considered “poor” quality [16,17].

### 3.4 Measurement of the outcome of interest

This systematic review and meta-analysis's main finding was to assess the magnitude of post-COVID-19 vaccine COVID-19 (SARS reinfection) among vaccinated individuals. The secondary outcome variable was the predictors of post COVID-19 vaccine SARS-CoV-2 infection, which were estimated using a pooled OR with 95 % confidence intervals (CIs). SARS-CoV-2 infection following COVID-19 vaccination was defined as the occurrence of individuals who tested positive for the COVID-19 virus after receiving the vaccine (Yes/No) [18].

### 3.5 Data synthesis and analysis

The statistical software STATA™ 17 was utilized to analyze the data imported from the Microsoft Excel spreadsheet. In the present meta-analysis, the “metaprop” command for STATA was employed to calculate the pooled estimate of the overall rate of SARS-CoV-2 infection following COVID-19 vaccination. A random-effects model was adopted in the meta-analysis to account for heterogeneity (I^2^ = 99.92 %, p = 0.000) with a 95 % confidence interval, and the results were visually presented in a forest plot. Additionally, funnel plots and the Egger's test were employed to assess the presence of publication bias. If the p-value of the Egger's test was less than 0.05, it was considered indicative of significant publication bias [19]. Sensitivity analyses and subgroup analysis were performed to identify possible moderators of the heterogeneity.

## 4 Results

### 4.1 Selection and identification of studies

**Fig. 1:**
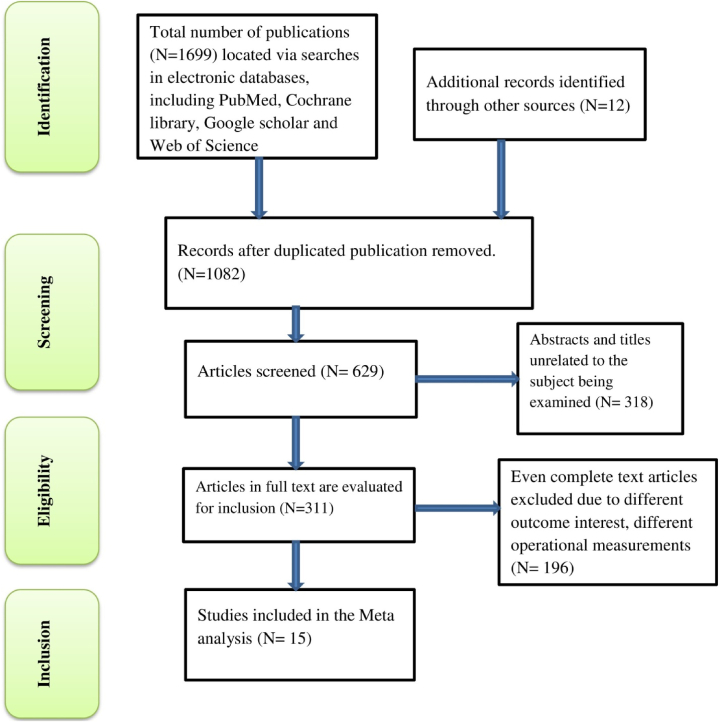
Flow chart diagram describing selection of studies included in the systematic review and meta-analysis using PRISMA checklist, 2024.

To identify relevant publications, various search techniques were employed, including African Journals Online, PubMed, Cochrane Review, EMBASE, Google Scholar, Web of Science, and Scopus. In total, 1711 articles were identified. Using the endnote reference manager, duplicate studies were removed, resulting in the elimination of 1082 articles. Subsequently, 318 articles were excluded based on their irrelevant titles and abstracts, leaving 311 papers for full-text screening. Out of these, 280 publications were eliminated: 196 due to inaccessibility of the full text and 75 because the desired outcome was not published. Ultimately, 15 studies that met the inclusion criteria were included in the analysis for this study (Fig. 1).

### 4.2 Characteristics of the included studies

A total of 342,598 individuals worldwide were included in this meta-analysis, with a 100 % response rate. All studies included in the analysis were published between 2021 and 2024 in international journals. Out of the total, 15 studies were included, with 6 being cross-sectional studies and the remaining being cohort studies. The sample sizes of the included studies ranged from 287 [20] to 160,719 [21] individuals. The studies were conducted in nine countries, specifically Ethiopia, India, United States, Israel, Saudi Arabia, France, Philippines, London, and Italy. The study with the lowest incidence was conducted in California, UCSD, with an incidence rate of 0.0012 % [12], while the highest incidence was observed in a study conducted in Saudi Arabia, with a rate of 17.9 % [13]. The quality of the studies was assessed using the Newcastle Ottawa scale and all studies being classified as having good quality. This critical evaluation aimed to assess the internal validity (systematic error) and external validity (generalizability) of the studies and minimize the risk of bias (Table 1).

**Table 1:** Summary of characteristics of included studies.

SN	Author	Year	Country	participants	Study design	Sample	Post-vaccine infection (N)	Quality assessment
1	Mohammed et al. [13]	2023	Saud-Arabia	Individuals	Cross-Sectional	694	124	Low risk
2	Keehner et al. [12]	2021	California	HCWs	Cohort	36,659	436	Low risk
3	Getahun et al. [2]	2023	Ethiopia	HCWs	Cross-Sectional	418	60	Low risk
4	Ronchini et al. [22]	2022	Milan, Italy	HCWs	Cohort study	2029	30	Low risk
5	Keehner et al. [12]	2021	California	HCWs	Cohort study	14,990	145	Low risk
6	Menni et al. [23]	2021	London (UK)	Individuals	Cohort study	103622	3106	Low risk
7	Vaishya et al. [24]	2021	India (Delhi)	HCWs	Retrospective study	3235	85	Low risk
8	Amit et al. [25]	2022	Israel	HCWs	Cross-Sectional	4081	22	Low risk
9	Kuodi et al. [26]	2022	Israel	Individuals	Survey	3572	637	Low risk
10	Bergwerk et al. [27]	2021	Israel	HCWs	Survey	1497	36	Low risk
11	Saade et al. [28]	2022	France	HCWs	Cohort study	6674	160	Low risk
12	Heudel et al. [29],	2022	France	Patients	Retrospective study	2391	39	Low risk
13	Selvaraj et al. [30]	2022	India	Individuals	Cross-Sectional	1729	450	Low risk
14	Kelly et al. [21]	2022	US, VHA	Patients	Cohort study	160719	20138	Low risk
15	Tan-Lim et al. [20]	2023	Philippines	Individuals	Cohort study	287	113	Low risk

## 5 Pooled incidence of SARS-CoV-2 infection after COVID -19 vaccinations

### 5.1 Prevention rate of COVID-19 vaccination

**Fig. 2:**
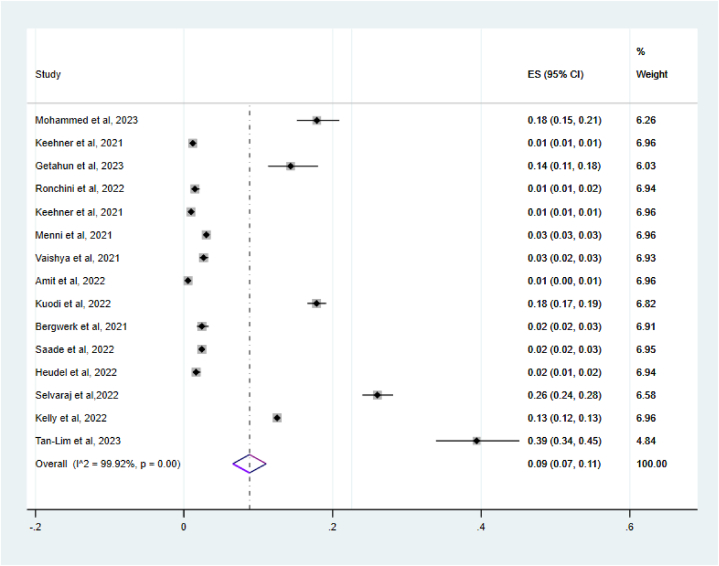
The overall pooled magnitude of SARS-CoV-2 infection of SARS-CoV-2 infection after COVID -19 Vaccinations among vaccinated individuals, 2024.

The introduction of vaccination reduced the overall attack rate of COVID-19 by 91 % after vaccination. The meta-analysis revealed that globally, the pooled incidence of COVID-19 symptoms following vaccination was 9 %, with a 95 % (7 %–11 %) (Fig. 2). Due to significant heterogeneity among the included studies (I^2^ = 99.92 %, p = 0.000), a random effects model was used. To assess the impact of individual studies on the overall effect, a leave-one-out sensitivity analysis was conducted, which revealed that no single study significantly influenced the overall incidence.

### 5.2 Sensitivity analysis

**Fig. 3:**
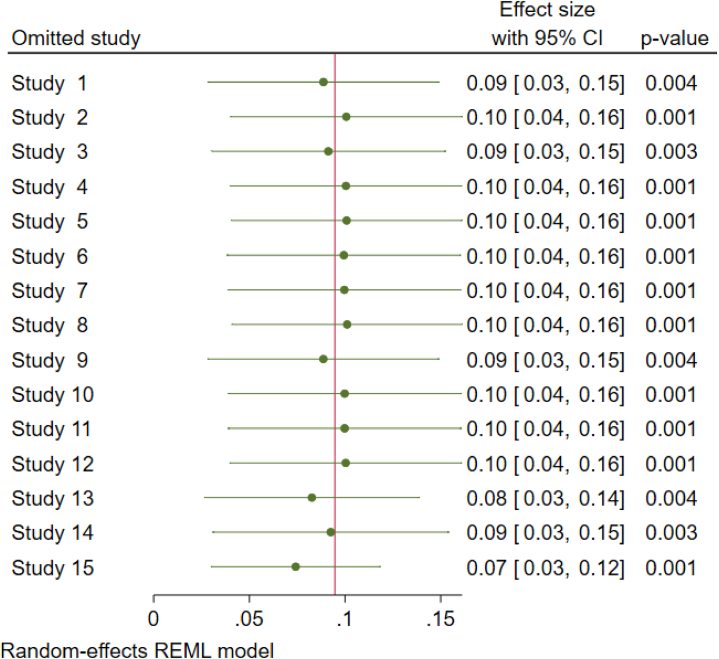
The pooled incidence of SARS-CoV-2 infection among vaccinated individuals after vaccination when one study omitted from the analysis a step at a time, 2024.

The researchers conducted a sensitivity analysis called leave-one-out, where they systematically excluded each study one at a time to see if it had a significant effect on the overall incidence of SARS-CoV-2 infection after COVID-19 vaccination. The results of the analysis, using a random effect model, indicated that none of the individual studies had a significant impact on the occurrence of SARS-CoV-2 infection among vaccinated individuals after vaccination (Fig. 3).

### 5.3 Subgroup analysis

**Fig. 4:**
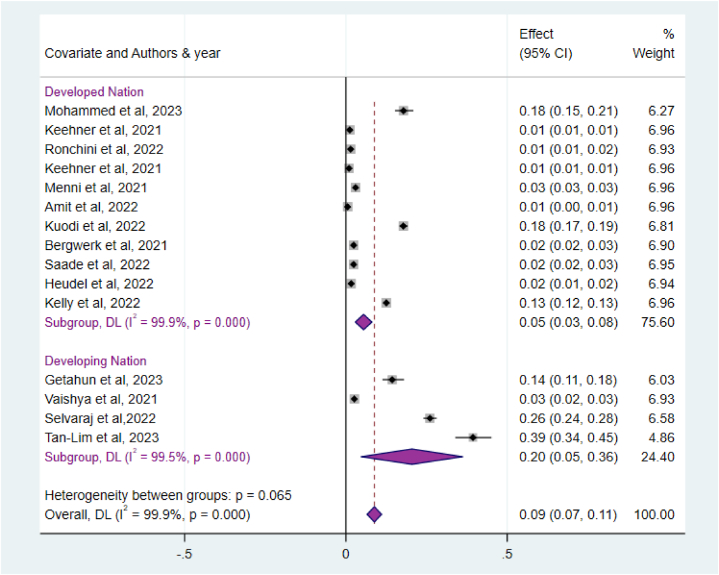
Sub-group analysis of studies included in meta-analysis depend on national GDP, 2024.

According to the classifications provided by the United Nations (UN), World Bank, and International Monetary Fund (IMF), countries were categorized into developed and developing nations [31]. For the subgroup analysis, studies conducted on specific groups were used, as indicated in (Fig. 4). The highest pooled estimated of SARS-CoV-2 infection after COVID -19 Vaccinations was seen among developing nations, 20 % (95 % CI: 5%–36 %).

### 5.4 Heterogeneity

The overall heterogeneity of SARS-CoV-2 infection after COVID -19 Vaccinations was Ι^2^ = 99.92 %, with P < 0.00 by use of the random effect model to adjust observed variability (Fig. 2).

### 5.5 Publication bias

**Fig. 5:**
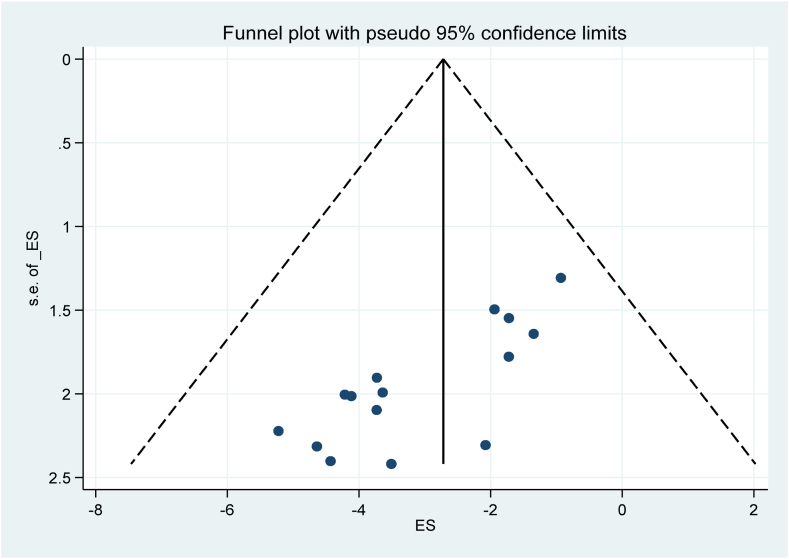
Study's publication bias, 2024.

The analysis conducted on the SARS-CoV-2 infection after COVID-19 vaccinations did not show any statistical evidence of publication bias. This was determined through the examination of a funnel plot and the use of the Egger regression test, with a significance level set at P > 0.05. The results of the Egger tests were not statistically significant, with P-values of 0.497, indicating no significant bias. Additionally, the funnel plot displayed a symmetrical distribution, suggesting the absence of small-study effects or publication bias (Fig. 5).

### 5.6 The predictors of SARS-CoV-2 infection after COVID-19 vaccinations

Due to inconsistent categorization (grouping) of the exposures in relation to the outcome, some of the components related to SARS-CoV-2 infection after COVID-19 Vaccinations in this review were quantitatively pooled while others were not. From the included studies, eight variables were taken to determine major determinants of SARS-CoV-2 infection after COVID -19 Vaccinations. Finally, two variables (age of participants and presence of comorbidity (chronic illness)) were identified as significant determinants of post COVID 19 vaccination's SARS-CoV-2 infection.

**Fig. 6:**
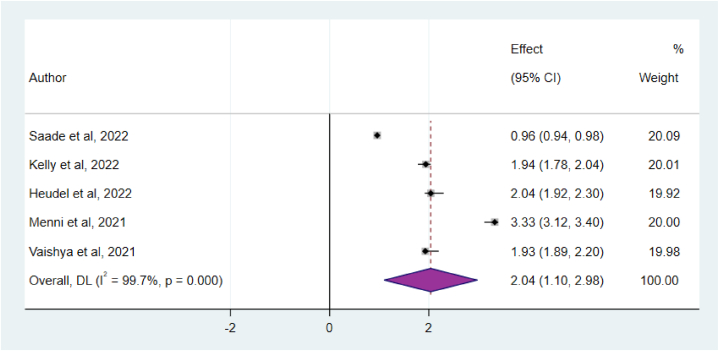
The association between age and post COVID-19 vaccination SARS-CoV-2 infection, 2024.

To examine the relationship between post COVID-19 vaccination SARS-CoV-2 infection and participant age, a total of five articles were analyzed. The results indicated that older participants had a higher risk of being infected with SARS-CoV-2 after vaccination compared to younger individuals. However, the remaining studies included in the analysis did not establish any association between the variables and the outcome. Overall, the findings of this study suggest that individuals aged fifty or older were more susceptible to experiencing post COVID-19 vaccination SARS-CoV-2 symptoms compared to younger respondents (OR = 2.04; 95%CI: 1.10–2.98) (Fig. 6)

**Fig. 7:**
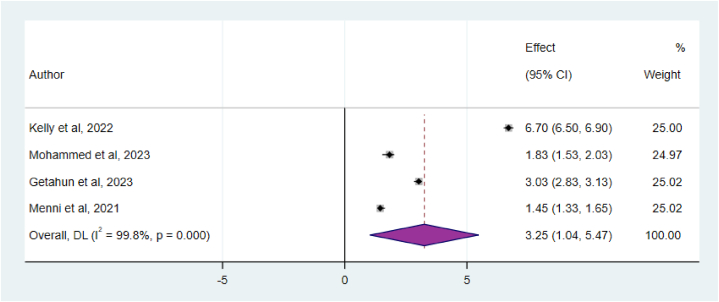
The association between comorbidities and post COVID-19 vaccination SARS-CoV-2 infection, 2024.

Furthermore, the analysis of data from four articles yielded a pooled odds ratio for the association between SARS-CoV-2 infection after COVID-19 vaccinations and comorbidities or chronic illness. The results of this study indicated that individuals with pre-existing chronic illnesses or comorbidities had a higher likelihood of developing SARS-CoV-2 infection after receiving the COVID-19 vaccine compared to those without any comorbidities (OR = 3.25; 95%CI: 1.04–5.47) (Fig. 7)

## 6 Discussion

Enhanced efforts to provide crucial post-vaccine care and implement mass COVID-19 strategies on a global scale are crucial in reducing human mortality. It is imperative that these services are readily accessible, particularly in low-income and middle-income countries. The review included fifteen studies from different countries, and all studies’ analyses used primary data. More than half (nine) of the included studies were follow up cohort studies. All of the studies included in this review had good quality (low risk). Since there was no literature on meta-analysis conducted on post-COVID-19 vaccination SARS-CoV-2 infection, comparable discussions might not be available in this discussion.

According to the findings of this systematic review and meta-analysis, the combined rate of SARS-CoV-2 infection following COVID-19 vaccination was estimated to be 9 % (95 % CI 7 %–11 %). Or, in other words the introduction of vaccination reduced the overall attack rate of COVID-19 by 91 % after vaccination. The finding is higher than some primary studies conducted in India [24], Israel [25,27] and London [23].

The subgroup analysis was desired to be performed due to the presence of a significant level of heterogeneity among the included studies. To check the sources of heterogeneity, subgroup analysis was done by using the economic status of the countries depending on United Nations (UN), World Bank, and International Monetary Fund (IMF) classification as ‘developed nations’ and ‘developing nation’ to assess the pooled prevalence of post-COVID-19 vaccination SARS-CoV-2 infection among vaccinated individuals. Accordingly, The highest pooled estimated of SARS-CoV-2 infection after COVID -19 Vaccinations was seen among developing nations, 20 % (95 % CI: 5%–36 %). This situation could be explained by various factors, including: (i) a potential increase in domestic transmission, leading to a higher rate of infection; and (ii) an increase in infections due to the resumption of international travel and trade as lockdown measures and travel restrictions are relaxed or lifted and the suboptimal handling of vaccines during transportation can result in the degradation or alteration of the drug, which can ultimately impact its effectiveness. Additionally, developed countries have the capacity to administer complete vaccinations according to the recommended schedule without any interruptions. This ability not only enhances immunity but also ensures the delivery of more effective vaccinations compared to developing nations [32,33].

In this systematic and meta-analytical analysis, the most common factors that predicted post vaccine COVID-19 symptoms were older age and comorbidity or chronic illness. Accordingly, individuals aged fifty or older were two time more susceptible to experiencing post COVID-19 vaccination SARS-CoV-2 symptoms compared to younger respondents (OR = 2.04; 95%CI: 1.10–2.98). This finding was supported by some primary studies [21,24,28]. This could be due to age-related decline in immune response: as individual's age, their immune system may not respond as robustly to the vaccine compared to younger individuals. This can result in a lower level of protection against the virus, making them more susceptible to breakthrough infections [34]. Variants of the virus: Emerging variants of the SARS-CoV-2 virus, such as the Delta variant, have shown increased transmissibility and the potential to partially evade the immunity provided by vaccines [35]. Older individuals, who may have received the vaccine earlier and have waning immunity, may be more susceptible to these variants. Additionally, older individuals often have underlying health conditions that can weaken their immune system or make them more vulnerable to infections. These conditions can increase the risk of breakthrough infections even after vaccination [36].

Furthermore, the analysis of data from four articles yielded a pooled odds ratio for the association between SARS-CoV-2 infection after COVID-19 vaccinations and comorbidities or chronic illness. The results of this study indicated that individuals with pre-existing chronic illnesses or comorbidities had a higher likelihood of developing SARS-CoV-2 infection after receiving the COVID-19 vaccine compared to those without any comorbidities (OR = 3.25; 95%CI: 1.04–5.47). This could be due to the increased susceptibility to COVID-19 reinfection in individuals with comorbidities may be attributed to their compromised immune response. Conditions like diabetes, heart disease, lung disease, and immunodeficiency disorders can weaken the immune system's ability to effectively respond to the virus, potentially making them more prone to reinfection. This justification was supported by different studies [37,38].

## 7 Implication of study

The study can contribute to the understanding of the safety profile of COVID-19 vaccines. Policymakers can use this knowledge to ensure public confidence in vaccination programs and address any concerns related to adverse events. Generally, the implications of this result would benefit governments, healthcare professionals, stakeholders, researchers, policymakers and implementers of health care, and the general public.

## 8 Conclusion and recommendation

The results of this systematic review and meta-analysis indicated that there was a high estimate of the pooled prevalence of post-COVID-19 vaccine SARS-CoV-2 infection among those who had received vaccinations globally. Policymakers should prioritize ongoing monitoring and surveillance of SARS infection rates among vaccinated individuals. This will help identify any potential breakthrough infections and assess the effectiveness of vaccination strategies. Policy makers and health professionals should continue to promote COVID-19 vaccination to ensure high vaccine coverage in the population. Encouraging individuals to receive the recommended vaccine doses can help enhance protection against reinfection.

## Ethics approval and consent to participate

The study conducted did not require ethical approval because it utilized secondary data from previously published literature.

## Consent for publication

Not applicable.

## Funding

No fund received for this study.

## Availability of data and materials

The datasets generated and/or analyzed during the current review study were publicly available at different searching engines (PubMed, Google scholar, Cochrane, Africa online etc.). In addition, those dataset used in this study submitted alongside with manuscript as additional file 1.

## CRediT authorship contribution statement

**Dagne Deresa Dinagde:** Writing – review & editing, Writing – original draft, Visualization, Validation, Software, Methodology, Data curation, Conceptualization. **Bekam Dibaba Degefa:** Writing – review & editing, Supervision, Methodology, Data curation. **Gemeda Wakgari Kitil:** Visualization, Supervision, Resources, Investigation, Data curation. **Gizu Tola Feyisa:** Writing – original draft, Validation, Resources, Methodology, Funding acquisition, Data curation. **Shambel Negese Marami:** Writing – review & editing, Validation, Project administration, Investigation, Data curation.

## Declaration of competing interest

The authors declare that they have no known competing financial interests or personal relationships that could have appeared to influence the work reported in this paper.

No conflict of financial interest.

## Abbreviations

SARS: Severe Acute Respiratory Syndrome
